# Does Laughter Predict Onset of Functional Disability and Mortality Among Older Japanese Adults? The JAGES Prospective Cohort Study

**DOI:** 10.2188/jea.JE20200051

**Published:** 2021-05-05

**Authors:** Yudai Tamada, Kenji Takeuchi, Chikae Yamaguchi, Masashige Saito, Tetsuya Ohira, Kokoro Shirai, Katsunori Kondo

**Affiliations:** 1 Department of Preventive Medicine, Nagoya University Graduate School of Medicine, Aichi, Japan; 2 Department of Community Health Nursing, Nagoya City University School of Nursing, Aichi, Japan; 3 Department of Social Welfare, Nihon Fukushi University, Aichi, Japan; 4 Department of Epidemiology, Fukushima Medical University School of Medicine, Fukushima, Japan; 5 Radiation Medical Science Center for Fukushima Health Management Survey, Fukushima Medical University, Fukushima, Japan; 6 Department of Social Medicine, Public Health, Graduate School of Medicine, Osaka University, Osaka, Japan; 7 Department of Social Preventive Medical Sciences, Center for Preventive Medical Sciences, Chiba University, Chiba, Japan; 8 Department of Gerontological Evaluation, Center for Gerontology and Social Science, National Center for Geriatrics and Gerontology, Aichi, Japan

**Keywords:** laughter, long-term care, death, cohort study, Japan

## Abstract

**Background:**

While laughter is broadly recognized as a good medicine, a potential preventive effect of laughter on disability and death is still being debated. Accordingly, we investigated the association between the frequency of laughter and onset of functional disability and all-cause mortality among the older adults in Japan.

**Methods:**

The data for a 3-year follow-up cohort including 14,233 individuals (50.3% men) aged ≥65 years who could independently perform the activities of daily living and participated in the Japan Gerontological Evaluation Study were analyzed. The participants were classified into four categories according to their frequency of laughter (almost every day, 1–5 days/week, 1–3 days/month, and never or almost never). We estimated the risks of functional disability and all-cause mortality in each category using a Cox proportional hazards model.

**Results:**

During follow-up, 605 (4.3%) individuals developed functional disability, identified by new certification for the requirement of Long-Term Care Insurance, and 659 (4.6%) deaths were noted. After adjusting for the potential confounders, the multivariate-adjusted hazard ratio of functional disability increased with a decrease in the frequency of laughter (*P* for trend = 0.04). The risk of functional disability was 1.42 times higher for individuals who laughed never or almost never than for those who laughed almost every day. No such association was observed with the risk of all-cause mortality (*P* for trend = 0.39).

**Conclusions:**

Low frequency of laughter is associated with increased risks of functional disability. Laughter may be an early predictor of functional disability later on in life.

## INTRODUCTION

Increasing functional disability, defined as difficulty in performing the activities of daily living, is a significantly important public health concern in rapidly aging societies worldwide.^1^ Particularly in Japan, one-fourth of its population of 127 million people is now aged ≥65 years.^2^ Furthermore, the number of people certified with functional disability has increased by nearly 1.4 times in the past decade, accounting for 17.3% of the Japanese population aged ≥65 years.^3^ Identifying the factors for preventing incident functional disability is a critical goal for super-aged societies, including Japan, because age-related functional disability negatively affects an individual’s health status, predicts mortality,^4^ and increases the healthcare costs associated with long-term care and hospital services.^5^^,^^6^

Laughter could potentially be regarded as medicine. Recently, an increasing number of studies have reported the beneficial effects of laughter on several health outcomes among older adults, such as on the cardiovascular functions and diseases and mental health.^7^^–^^11^ However, studies assessing the association between laughter and functional disability and mortality, while considering the individuals’ socioeconomic background, have not been reported. The frequency of laughter can vary according to an individual’s socioeconomic status,^12^ which is associated with the late-life health trajectories.^13^ The socioeconomic status can be considered a common cause of the association between laughter and health outcomes. Therefore, by targeting a large general population of community-dwelling older adults, this prospective cohort study aimed to test the hypothesis that low frequency of laughter is associated with a higher risk of onset of functional disability and all-cause mortality when the socioeconomic status is taken into consideration.

## METHODS

### Study sample

This study was based on the cohort data from the Japan Gerontological Evaluation Study (JAGES),^14^ which is an ongoing longitudinal study investigating the factors associated with health and well-being in the community-dwelling adults aged ≥65 years who could independently perform the physical and cognitive activities of daily living. Functional independency was defined as not being certified for Japan’s national Long-term Care Insurance system. We used the data of the 2013 wave (from October to December). In the 2013 wave, self-reported questionnaires were mailed to 193,694 community-dwelling elderly adults aged ≥65 years in 30 municipalities; of these, 137,736 individuals responded to the survey (response rate = 71.1%). The questionnaire comprised basic questions and five modules that covered different topics, as follows: module A, nursing care and medical care and lifestyles; module B, oral hygiene, optimism, and subjective health; module C, social capital and history of abuse; module D, subjective quality of life, sleep, and cognitive function; and module E, physical activity. Of the respondents, 21,377 individuals in 23 municipalities in 9 (out of 47) prefectures responded to the basic questions and module B, including questions about laughter, in the questionnaire of the JAGES. Of the eligible sample of 21,377 individuals, 20,714 were successfully associated with the administrative records in 2016, corresponding to a follow-up rate of 96.9%. After excluding 6,481 participants with missing information regarding the frequency of laughter (*n* = 958), annual household income (*n* = 3,191), medical history (*n* = 878), and survey questions on other covariates used in the analysis (*n* = 1,454), we finally analyzed the data of 14,233 participants (men, 7,162; women, 7,071).

### Outcomes

The outcomes of the present study were the onset of functional disability and all-cause mortality obtained from the municipal and national databases. The onset of functional disability was determined when an individual was newly certified for Long-term Care Insurance level 2–5,^15^^,^^16^ which is based on a multistep assessment of functional and cognitive impairments by a qualified investigator and on comments from the family physician.^17^ Information regarding the onset of mortality was obtained from the administrative databases of the national Long-term Care Insurance registers. These definitions were used in previous epidemiological studies.^18^^,^^19^

### Exposure

The daily frequency of laughter was measured based on the response to the following standard single-item question: “How often do you laugh out loud?” The possible item answers were as follows: almost every day, 1–5 days per week, 1–3 days per month, or never or almost never. The 1-year test-retest reliability of the question was reported in a previous study^20^; subsequently, regional and seasonal differences in the daily frequency of laughter among the Japanese men and women were not observed. This item had been used in several previous studies.^8^^,^^9^^,^^12^^,^^21^

### Covariates

We included a wide range of covariates in the analyses as potential confounders based on prior literature.^8^^,^^9^^,^^12^^,^^18^^,^^21^ Information on sex, age, hypertension, diabetes mellitus, smoking habit, alcohol intake, family structure, social participation, depressive symptoms, cognitive function, instrumental activities of daily living (IADL), educational attainment, and equivalent income was obtained from a self-administered questionnaire. Smoking habit and alcohol intake were classified into the following three categories: current, ever, and never. We considered the respondents who answered “Yes” to the question, “Have you ever been diagnosed with hypertension or diabetes mellitus?” as participants with hypertension or diabetes mellitus, respectively. Family structure was assessed through two questions, one related to marital status and the other to number of people living together. The marital status question provided five answer categories (married, bereaved, divorced, never married and other). According to the responses to these questions, family structure was classified into four groups: alone, ≥2 without partner, ≥2 with partner, or ≥2 with no information about marital status. Social participation was defined as the person’s involvement in social activities (eg, volunteer group, sports group or club, leisure activity group, senior citizen club, neighborhood association or residents’ association, study or cultural group, nursing care prevention or health building, teaching skills or passing on experiences to others, local events). We defined the participants who engaged in one or more of the social activities more than once per week as socially active. To assess the depressive symptoms, we used the 15-item Geriatric Depression Scale; the participants were categorized into the following two groups based on the scores: not depressed (0–4 points) and depressed (≥5 points).^22^^,^^23^ Cognitive function was assessed through three questions (part of the Kihon Check-list,^24^ a basic function checklist in Japanese): First, Do your family or your friends point out your memory loss? Second, Do you make a call by looking up phone numbers? Third, Do you find yourself not knowing today’s date? Participants are asked to respond either “negative” (score: 1) or “positive” (score: 0). We divided the participants into the following two groups based on the scores: Decline (1–3 points) and Normal (0 point). Our assessment of IADL was based on a five-item subscale of the Tokyo Metropolitan Institute of Gerontology Higher Competence Scale.^25^ We categorized those who had difficulty with at least one item as ‘dependent’; others were categorized as ‘independent.’ Attainment of education and annual equivalent income served as indicators of the socioeconomic status. Attainment of education was evaluated based on the self-reported history of education and was classified into two categories (≤9 years and ≥10 years). The equivalent income was divided into nine categories (≤$14,900, $15,000–19,900, $20,000–24,900, $25,000–29,900, $30,000–34,900, $35,000–39,900, $40,000–45,900, $45,000–49,900, and ≥$50,000).

### Statistical analysis

For the demographic characteristics, summary statistics were constructed using frequencies for categorical variables. Linear trends regarding the frequencies of risk factors according to the frequency of laughter categories were tested using logistic regression analysis. Cox proportional hazards model was used to estimate the crude and adjusted hazard ratios (HRs) and their 95% confidence intervals (CIs) for the onset of functional disability and all-cause mortality according to the frequency of laughter. In multivariate adjustment, all covariates (sex, age, hypertension, diabetes mellitus, smoking habit, alcohol intake, marital status, social participation, depressive symptoms, educational attainment, and equivalent income) were included. All statistical analyses were performed using the International Business Machines Corporation Statistical Package for the Social Sciences (SPSS) version 25 statistical software (SPSS, Inc.; Chicago, IL, USA), and two-sided *P*-values <0.05 were considered statistically significant in all cases.

### Ethical issues

Our study protocol and informed consent procedure were approved by the Ethics Committee on Research of Human Subjects at Nihon Fukushi University (August 6, 2013, No 13-14).

## RESULTS

Table 1 shows the baseline characteristics of the study population according to the frequency of laughter. The likelihood of being female, being socially active, and having 10 years or more of education increased gradually with the increasing frequency of laughter. The likelihood of having been diagnosed with diabetes mellitus, being with cognitive decline, being dependent in IADL and being depressed decreased gradually with the increasing frequency of laughter. The frequency of age, smoking habit, alcohol intake, family structure, and equivalent income categories were significantly different across the frequency of laughter categories.

**Table 1.** Baseline characteristics of the study population by the frequency of laughter

	Almost every day (*n* = 6,120)		1–5 days per week (*n* = 5,440)		1–3 days per month (*n* = 1,639)		Never or Almost never (*n* = 1,034)		*P* for trend

	*n*	%	*n*	%	*N*	%	*N*	%	
Women	3,417	55.8	2,700	49.6	603	36.8	351	33.9	<0.001
Age, years									<0.001^a^
65–69	1,925	31.5	1,683	30.9	468	28.6	262	25.3	
70–74	2,078	34.0	1,632	30.0	465	28.4	293	28.3	
75–79	1,245	20.3	1,198	22.0	387	23.6	220	21.3	
80–84	616	10.1	651	12.0	220	13.4	155	15.0	
≤85	256	4.2	276	5.1	99	6.0	104	10.1	
Hypertension									0.537
Diagnosed	2,779	45.4	2,487	45.7	745	45.5	483	46.7	
Diabetes mellitus									<0.001
Diagnosed	827	13.5	740	13.6	254	15.5	189	18.3	
Smoking habit									<0.001^a^
Current	571	9.3	554	10.2	229	14.0	175	16.9	
Ever	903	14.8	998	18.3	363	22.1	229	22.1	
Never	4,646	75.9	3,888	71.5	1,047	63.9	630	60.9	
Alcohol Intake									<0.001^a^
Current	2,138	34.9	1,995	36.7	677	41.3	393	38.0	
Ever	262	4.3	282	5.2	113	6.9	74	7.2	
Never	3,720	60.8	3,163	58.1	849	51.8	567	54.8	
Family structure									<0.001^a^
Alone	679	11.1	890	16.4	314	19.2	261	25.2	
≥2 without partner	793	13.0	722	13.3	189	11.5	147	14.2	
≥2 with partner	4,608	75.3	3,787	69.6	1,118	68.2	614	59.4	
≥2 without information about marital status	40	0.7	41	0.8	18	1.1	12	1.2	
Social participation									<0.001
Active	2,186	35.7	1,816	33.4	368	22.5	151	14.6	
Depressive symptoms									<0.001
Depressed	872	14.2	1,416	26.0	672	41.0	618	59.8	
Cognitive function									<0.001
Decline	1,871	30.6	1,905	35.0	690	42.1	528	51.1	
Instrumental activities of daily living									<0.001
Dependent	993	16.2	1,018	18.7	391	23.9	332	32.1	
Attainment of education									<0.001
≥10 years	3,835	62.7	3,288	60.4	1,004	61.3	538	52.0	
Equivalent income (10,000$, 1$ = 100 yen)									<0.001^a^
≤1.49	1,439	23.5	1,476	27.1	520	31.7	425	41.1	
1.50–1.99	1,285	21.0	1,296	23.8	406	24.8	228	22.1	
2.00–2.49	1,181	19.3	979	18.0	303	18.5	158	15.3	
2.50–2.99	364	5.9	330	6.1	72	4.4	49	4.7	
3.00–3.49	643	10.5	506	9.3	107	6.5	51	4.9	
3.50–3.99	398	6.5	308	5.7	93	5.7	43	4.2	
4.00–4.49	156	2.5	99	1.8	27	1.6	17	1.6	
4.50–4.99	193	3.2	148	2.7	33	2.0	22	2.1	
≥5.00	461	7.5	298	5.5	78	4.8	41	4.0	

^a^Tested using chi-square test.

**Figure 1.**
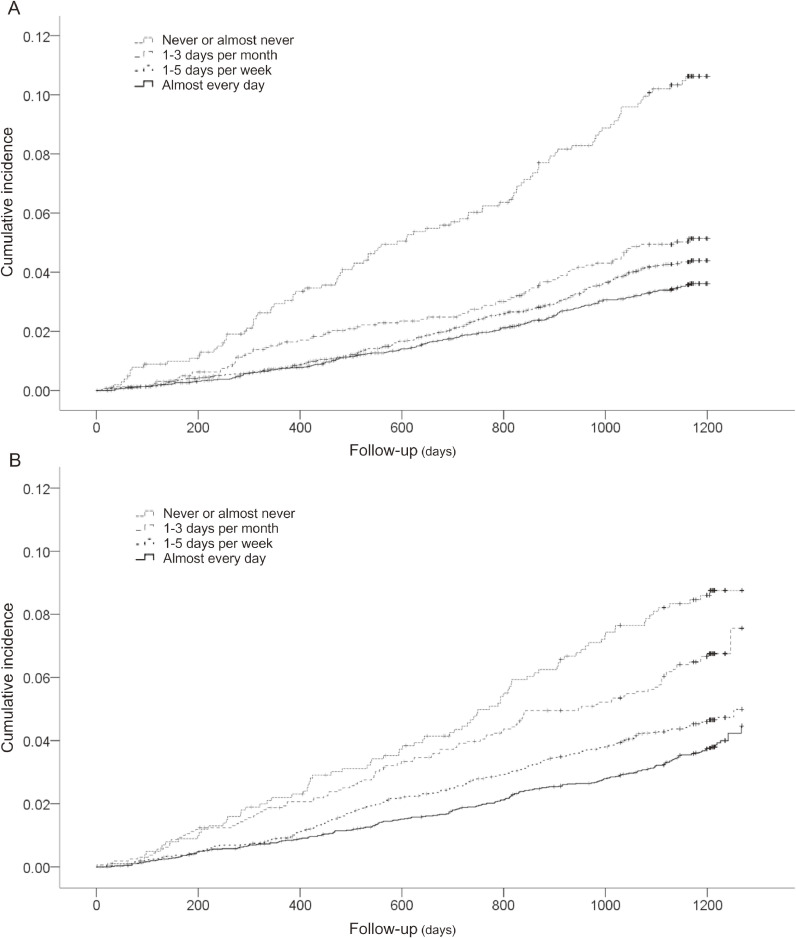
Kaplan-Meier curves showing cumulative incidence of functional disability (A) and all-cause mortality (B) according to the frequency of laughter

During follow-up (median, 3.3 years), 605 (4.3%) individuals developed functional disability and 659 (4.6%) deaths were noted. The all-cause mortality and functional disability rates were compared according to the daily frequency of laughter using the Kaplan-Meier method. Functional disability and all-cause mortality were more commonly observed among participants with a low frequency of laughter (log-rank test, *P* < 0.001, Figure 1A and log-rank test, *P* < 0.001, Figure 1B, respectively).

Table 2 shows the results of Cox proportional hazards analysis for the association of the frequency of laughter and functional disability and all-cause mortality. In the crude model, significantly inverse associations between the frequency of laughter and functional disability (*P* for trend <0.001) and all-cause mortality (*P* for trend <0.001) were observed. These inverse associations remained significant after adjusting for sex and age (functional disability, *P* for trend <0.001; all-cause mortality, *P* for trend = 0.001). After adjusting for the abovementioned covariates, the multivariate-adjusted HR of functional disability increased with a decrease in the frequency of laughter (*P* for trend = 0.04). The risk of developing functional disability was 1.42 times higher for individuals who laughed never or almost never than for those who laughed almost every day (95% CI, 1.10–1.85). However, no such association was observed with the risk of all-cause mortality (*P* for trend = 0.39).

**Table 2.** Likelihood of onset of functional disability and all-cause mortality according to the frequency of laughter

	Almost every day (*n* = 6,120)	1–5 days per week (*n* = 5,440)	1–3 days per month (*n* = 1,639)	Never or Almost never (*n* = 1,034)	*P* for trend
Functional disability, *n*	206	223	78	98	
Crude HR (95% CI)	1.00 (reference)	1.23 (1.02–1.49)	1.45 (1.12–1.88)	2.98 (2.34–3.79)	<0.001
Adjusted HR (95% CI)^a^	1.00 (reference)	1.13 (0.94–1.37)	1.22 (0.94–1.59)	2.14 (1.68–2.74)	<0.001
Adjusted HR (95% CI)^b^	1.00 (reference)	1.04 (0.86–1.26)	0.97 (0.74–1.27)	1.42 (1.10–1.85)	0.039
All-cause mortality, *n*	226	244	104	85	
Crude HR (95% CI)	1.00 (reference)	1.22 (1.02–1.47)	1.75 (1.38–2.20)	2.29 (1.79–2.94)	<0.001
Adjusted HR (95% CI)^a^	1.00 (reference)	1.10 (0.92–1.32)	1.35 (1.07–1.70)	1.52 (1.18–1.96)	<0.001
Adjusted HR (95% CI)^b^	1.00 (reference)	1.03 (0.86–1.24)	1.13 (0.89–1.44)	1.08 (0.83–1.41)	0.389

CI, confidence interval; HR, hazard ratio.

^a^Adjusted for sex and age.

^b^Adjusted for sex, age, hypertension, diabetes mellitus, smoking, alcohol intake, family structure, social participation, depressive symptoms, cognitive function, instrumental activities of daily living, educational attainment, and equivalent income.

## DISCUSSION

To the best of our knowledge, this is the first study to comprehensively examine the association between laughter and functional disability and all-cause mortality after carefully controlling for the potential confounders, such as the socioeconomic status. The present prospective cohort study of community-dwelling Japanese older adults revealed an inverse association between the daily frequency of laughter and onset of functional disability, indicating that participants with a lower frequency of laughter were at higher risk of the onset of functional disability. Particularly, laughing never or almost never could increase the risk of functional disability by nearly 50%. In this study, approximately one-fifth of the participants laughed less than once per week; hence, it is reasonable to hypothesize that public health efforts regarding the dissemination of information on the importance of laughter to reduce the future incidence of functional disability predicting mortality among the older adults are warranted.

While published reports indicating the association between the frequency of laughter and functional disability are not currently available, several previous reports revealed that the daily frequency of laughter was associated with the prevalence and incidence of cardiovascular diseases,^8^^,^^21^ which constitute the second leading cause of functional disability in Japan.^26^ Based on our present results being in line with these previous findings, we provide valuable new evidence that the low frequency of laughter itself contributes to the development of functional disability, independent of the established confounders.

There are several plausible mechanisms underlying the association between laughter and functional disability among the older adults. First, laughter might produce physiological changes in various systems of the body,^27^ such as improvement of the immune function^28^ and stimulation of circulation.^29^ In turn, a low frequency of laughter can trigger functional impairments. Second, a high frequency of laughter may be a marker of positive emotions in daily life, which is associated with lower functional limitations.^30^ Moreover, laughter-related positive emotions are able to downregulate the cardiovascular aftereffects of negative emotions, which can serve as a buffer against functional disability.^31^ Finally, laughter can play a role in buffering the effects of stress. For example, stimulated and spontaneous laughter is reported to decrease salivary cortisol level, a biomarker of stress.^32^^,^^33^ Thus, individuals with a higher frequency of laughter may cope more effectively with stress than individuals with a lower frequency of laughter, which may moderate the adverse effects of stress on the individuals’ physical health.

Regarding all-cause mortality, our study revealed that age- and sex-adjusted HR of all-cause mortality increased with a decrease in the daily frequency of laughter, but this inverse association was insignificant after adjusting for all covariates. Meanwhile, a recent previous study^21^ reported a significant association between the daily frequency of laughter and all-cause mortality. This discrepancy is possibly attributed to the differences in the study settings (nine prefectures covering a wide area in Japan vs one prefecture), participants (a general population of community-dwelling older adults vs community-based annual health checkup examinees), and controlling for the confounding effects of the socioeconomic status (adjusted vs unadjusted). The present study attempted to reduce the degree of selection bias and potential confounding effects as much as possible. In contrast, both studies included a limited number of mortality events during relatively short periods of time, namely 3–5 years. Thus, further long-term follow-up studies are warranted to elucidate the association between the daily frequency of laughter and onset of mortality.

The primary strengths of the present study are its prospective cohort design, large sample size, population-based sampling, and control for potential confounding factors. In contrast, a limitation of the study was that we evaluated the daily frequency of laughter using a single-item self-reported question. The perceived frequency of laughter may be different from the actual frequency; hence, it may be plausible that less healthy individuals are more likely to not report their frequency of laughter, possibly leading to an underestimation of the association between laughter and health outcomes. Additionally, it is unclear whether laughter itself can prevent the onset of functional disability and mortality. Therefore, further studies are required to precisely identify the causal inference using observational data^34^ because random assignment of the daily frequency of laughter and long-term follow-up of the randomized participants to collect the data on number of onset events are difficult in the real-world setting.

In conclusion, the present study revealed that community-dwelling older Japanese who do not laugh much in daily life are at a higher risk of the onset of functional disability, suggesting that the frequency of laughter is potentially considered an early indicator of late-life functional disability.
